# Draft Genome Sequence of *Ochrobactrum intermedium* Strain SA148, a Plant Growth-Promoting Desert Rhizobacterium

**DOI:** 10.1128/genomeA.01707-16

**Published:** 2017-03-02

**Authors:** Feras F. Lafi, Intikhab Alam, Rene Geurts, Ton Bisseling, Vladimir B. Bajic, Heribert Hirt, Maged M. Saad

**Affiliations:** a King Abdullah University of Science and Technology (KAUST), Biological and Environmental Sciences and Engineering Division (BESE), Thuwal, Kingdom of Saudi Arabia; b Computational Bioscience Research Center (CBRC), King Abdullah University of Science and Technology (KAUST), Thuwal, Kingdom of Saudi Arabia; c Department of Plant Sciences, Laboratory of Molecular Biology, Wageningen University, Wageningen, The Netherlands

## Abstract

*Ochrobactrum intermedium* strain SA148 is a plant growth-promoting bacterium isolated from sandy soil in the Jizan area of Saudi Arabia. Here, we report the 4.9-Mb draft genome sequence of this strain, highlighting different pathways characteristic of plant growth promotion activity and environmental adaptation of SA148.

## GENOME ANNOUNCEMENT

The DARWIN21 project (http://www.darwin21.net/) is an initiative established with the aim of expanding our knowledge on microbial life in deserts. A diverse number of bacterial species were isolated from both soil and plants and were characterized for their potential application in modern agriculture. Within the DARWIN21 collection, diverse bacterial species show enhanced growth of the model plant *Arabidopsis thaliana* under stress conditions (1, 2). A number of bacterial *Ochrobactrum* species were isolated on the basis of their plant growth-promoting trait. *Ochrobactrum* species are widespread in the environment and can colonize a wide variety of habitats, including soil, plants, animals, and humans (3). In an attempt to decipher the genetic basis of the plant growth-promoting activity of a member of the *Ochrobactrum* species, the genome of strain SA148 was sequenced.

SA148 strain was isolated from the soil samples collected in the Jizan region (16°56.475′N, 42°36.694′E) in the Kingdom of Saudi Arabia. Based on the 16S rRNA gene sequences, the SA148 strain is closely related (99% gene similarity) to *Ochrobactrum intermedium* strain LMG3301 (accession no. NR_026039.1) (1). The genomic DNA of the SA148 strain was extracted using the Qiagen DNeasy blood and tissue kit, according to the manufacturer’s protocol. The DNA was then sequenced using paired-end Illumina MiSeq, and the library was constructed as described previously (4). Contig assembly was done with SPAdes assembler version 3.6 (5), with a 1-kb contig cutoff size. *De novo* assembly of MiSeq reads for strain SA148 resulted in 38 contigs with a total length of 4,918,899 bp, a mean contig size of 129,445 bp, an *N*_50_ of 419,572 bp, *L*_50_ reached with five contigs, and a genome G+C content of 57.5%. MegaBLAST (6) searches of strain SA148 concatenated genomes against the NCBI reference genome database (http://www.ncbi.nlm.nih.gov/genome/) revealed that the closest relative genomes to strain SA148 was *O. intermedium* strain LMG3301, with sequence coverages of 50% and 35% of chromosomes I and II (accession no. ACQA0100000.1) and similarities of 98% and 99%, respectively. The annotation of strain SA148 was carried out using the default INDIGO pipeline (7), with the exception of open reading frame (ORF) prediction by FragGeneScan (8). SA148 has 3,714 ORFs, three rRNA, 51 tRNA, and 64 noncoding RNA (ncRNA). The annotation predicted a number of enzymes, such as glyphosate resistance enzyme 3-phosphoshikimate 1-carboxyvinyltransferase (EC 2.5.1.19) first identified in *Ochrobactrum anthropi* (9), which is targeted by the herbicide glyphosate. We also detected mannitol-1-phosphate 5-dehydrogenase (EC 1.1.1.17) (gene *mtlD*) as a part of an osmoprotectant synthesis pathway that can be utilized to improve stress tolerance against temperature fluctuation (10). Moreover, a number of genes related to growth-promoting activity, including gene-coding clusters for phosphate solubilization with six genes for pyrroloquinoline quinone synthesis (PQQ) (EC 1.1.5.2) and glucose 1-dehydrogenase (GCD) (EC 1.1.5.2) were found. The genome analysis highlights the presence of 3-phytase (EC 3.1.3.8) (11) and acid phosphatase (EC 3.1.3.2) (12), two enzymes that aid plants during phosphate starvation.

### Accession number(s).

The genome of *Ochrobactrum intermedium* SA148 was deposited at DDBJ/EMBL/GenBank under the accession no. LWEA00000000. The version described in this paper is the first version, LWEA01000000.
